# The role of the proteasome in AML

**DOI:** 10.1038/bcj.2016.112

**Published:** 2016-12-02

**Authors:** C M Csizmar, D-H Kim, Z Sachs

**Affiliations:** 1 Department of Medicinal Chemistry, University of Minnesota, Minneapolis, MN, USA; 2 Department of Biochemistry, Molecular Biology and Biophysics, University of Minnesota, Minneapolis, MN, USA; 3 Department of Medicine, Division of Hematology, Oncology and Transplantation, University of Minnesota, Minneapolis, MN, USA

## Abstract

Acute myeloid leukemia (AML) is deadly hematologic malignancy. Despite a well-characterized genetic and molecular landscape, targeted therapies for AML have failed to significantly improve clinical outcomes. Over the past decade, proteasome inhibition has been demonstrated to be an effective therapeutic strategy in several hematologic malignancies. Proteasome inhibitors, such as bortezomib and carfilzomib, have become mainstays of treatment for multiple myeloma and mantle cell lymphoma. In light of this success, there has been a surge of literature exploring both the role of the proteasome and the effects of proteasome inhibition in AML. Pre-clinical studies have demonstrated that proteasome inhibition disrupts proliferative cell signaling pathways, exhibits cytotoxic synergism with other chemotherapeutics and induces autophagy of cancer-related proteins. Meanwhile, clinical trials incorporating bortezomib into combination chemotherapy regimens have reported a range of responses in AML patients, with complete remission rates >80% in some cases. Taken together, this preclinical and clinical evidence suggests that inhibition of the proteasome may be efficacious in this disease. In an effort to focus further investigation into this area, these recent studies and their findings are reviewed here.

## Introduction

Acute myeloid leukemia (AML) is a lethal hematologic malignancy characterized by the neoplastic accumulation of immature myeloid cells.^1^ The standard of care chemotherapy regimen for AML was established over 30 years ago and remains largely unchanged today.^2^ This regimen, consisting of cytarabine and an anthracycline, achieves a complete remission (CR) in up to 85% of adults who are 60 years of age or younger; however, most patients will relapse within 3 years.^2^ In spite of salvage options – including additional chemotherapy and allogeneic hematopoietic stem cell transplantation – the prognosis for patients who relapse is uniformly poor, with 5-year overall survival (OS) probabilities ranging from 4 to 46%.^2^ In elderly patients (>60 years), the prognoses for both primary and relapsed AML are even worse. Finally, prognosis is dismal for those who cannot tolerate standard induction chemotherapy, with a median survival of only 5–10 months and OS of <5%.^2^ Thus, there is a clear and emergent need for the development of new therapeutic approaches for AML.

One promising molecular target is the proteasome, a large multimeric protein complex that degrades unneeded or damaged proteins.^3, 4^ As such, the proteasome has an integral role in a variety of cellular processes, including cell survival, cell signaling and cell-cycle progression.^4, 5^ Malignant cells are highly dependent on increased protein production and degradation, suggesting that they would be sensitive to proteasome inhibition.^6, 7, 8^ Indeed, proteasome inhibition is a mainstay of therapy in lymphoid malignancies. Proteasome inhibitors, such as bortezomib and carfilzomib, are now incorporated into standard of care regimens for most patients with multiple myeloma (MM) and other plasma cell neoplasms, and this approach has yielded significantly improved clinical responses and OS for these patients.^9^ Proteasome inhibition has also shown efficacy in the initial treatment of mantle cell lymphoma (MCL)^10^ and in the relapsed/refractory setting for other non-Hodgkin lymphomas, such as follicular lymphoma.^11, 12^

Several pre-clinical and early stage clinical trials investigating the role of the proteasome and proteasome inhibition in AML have shown promising results. In this review, we discuss these studies and their findings.

## Molecular effects of proteasome inhibition in AML

### Constitutive nuclear factor κB signaling is supported by the proteasome

**Figure 1:**
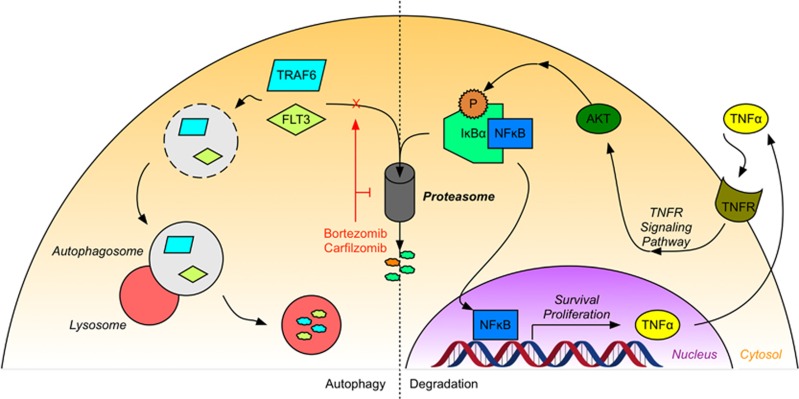
The proteasome has several roles in AML. The primary function of the proteasome is the proteolytic degradation of ubiquitinated proteins. In AML, phosphorylation of IκBα targets this regulatory protein for ubiquitination and proteasomal degradation. Degradation of IκBα liberates NF-κB, allowing this transcription factor to translocate to the nucleus and promote the expression of pro-survival and proliferative gene products, including TNFα. Among other actions, TNFα binds to the tumor necrosis factor receptor and drives an autocrine signaling pathway, promoting further IκBα phosphorylation and creating a positive-feedback loop that reinforces NF-κB activity. Inhibition of proteasome activity by agents such as bortezomib or carfilzomib both disrupt this cycle, leading to cell death, and also induce other cellular mechanisms of protein degradation, such as autophagy. AML cells treated with bortezomib can sequester cytosolic proteins within membrane-bound vesicles called autophagosomes. These proteins, including the cancer-related proteins FLT3 and TRAF6, are then delivered to the lysosome for oxidative degradation.

Nuclear factor κB (NF-κB) is a transcription factor that promotes cell survival and proliferation and has been implicated in the pathogenesis of numerous malignancies.^13^ In AML, NF-κB is constitutively active in leukemic stem cells (LSCs), but not in normal hematopoietic progenitor cells.^13^ This constitutive NF-κB activity is supported by autocrine signaling via tumor necrosis factor α (TNF-α), which directs the proteasome-mediated degradation of the NF-κB inhibitor IκBα, thereby liberating cytosolic NF-κB.^14^ As NF-kB promotes TNF-α expression, a positive-feedback loop is created between NF-κB and TNF-α, promoting cell survival and progression of the leukemia (Figure 1).^14^

This NF-κB/TNF-α feedback loop – and the survival of LSCs – is highly dependent upon the proteasomal degradation of the regulatory protein IκBα. Under normal, unstimulated circumstances, IκBα binds to NF-κB and sequesters it in the cytosol, preventing NF-κB from binding its gene targets within the nucleus. TNF-α signaling drives the phosphorylation of serine residues 32 and 36 on IκBα, leading to ubiquitination and proteasomal degradation.^13, 14^ This liberates NF-κB and allows the complex to translocate to nucleus where it can direct the expression of target genes, including TNFα. The importance of the proteasome in this process is underscored by early studies of the effects of proteasome inhibition on NF-κB signaling. For example, treating AML cells with the proteasome inhibitor MG-132 increased the amount of phosphorylated IκBα, leading to a strong inhibition of NF-κB activity, decreased expression of NF-κB gene targets and induction of apoptosis in AML cells (including LSCs).^13^ Importantly, normal hematopoietic stem cells (HSCs), which are not reliant upon NF-κB activity, were spared from this effect.^13^ These results were amplified when MG-132 was combined with the anthracycline idarubicin. Treating primary AML cells with this combination ablated LSCs and prevented tumor engraftment in NOD/SCID xenogeneic mouse models, while the ability of normal HSCs to proliferate was not significantly impaired.^15^

Because of the poor stability, low bioavailability and relatively low specificity of MG-132, more recent studies have used bortezomib to modulate NF-κB signaling in AML. Like MG-132, bortezomib treatment of AML cell lines and primary blasts increases the abundance of phosphorylated IκBα, reduces NF-κB activity, and triggers apoptosis via permeabilization of the mitochondrial membrane, release of cytochrome C, and cleavage of caspases 3, 8 and 9.^16, 17^ When primary AML cells from 28 patients were treated with either bortezomib or conventional chemotherapeutics (doxorubicin, cytarabine, or fludarabine), bortezomib was found to be the most potent of these agents (IC_50_ values of 5–10 nM) while exhibiting less toxicity than doxorubicin towards normal residual lymphocytes.^17^

### Proteasome inhibition is synergistic with other chemotherapeutics

Bortezomib has been shown to exhibit synergistic anti-AML cytotoxicity (against both cell lines and primary samples) *in vitro* when co-administered alongside a variety of other chemotherapeutics. As anthracyclines (for example, doxorubicin, daunorubicin and idarubicin) are already mainstays of AML therapy, there have been several studies investigating the effects of co-treatment with these agents.^15, 18, 19, 20^ By and large, the synergistic effects observed in this setting appear to be due to enhanced inhibition of NF-κB signaling and, to a lesser degree, activation of p53.^15^ Co-administration with histone deacetylase inhibitors (belinostat, panobinostat and valproic acid) also perturbs NF-κB, while inhibition of AKT signaling and down-regulation of anti-apoptotic proteins such as Bim, Bcl-2 and XIAP (X-linked inhibitor of apoptosis protein) are also observed.^21, 22, 23, 24, 25^

Bortezomib co-treatment alongside nucleoside analogs (for example, cytarabine, azacitidine and decitabine)^26, 27, 28, 29, 30^ has also been studied. This approach is further supported by evidence that bortezomib treatment indirectly upregulates transcription of DNA methyl-transferase 1 (DNMT1), and azacitidine has been shown to inhibit the activity of DNMT enzymes.^29^ In addition, bortezomib has been shown to upregulate the expression of the inhibitory microRNA precursor *miR-29b*, and because patients with elevated *miR-29b* expression have shown promising responses to decitabine, this combination regimen was also tested clinically.^30^

Taken together, this data suggests that proteasome inhibition remains an efficacious therapeutic mechanism independent of other approaches. Finally, bortezomib retains its activity regardless of p53 status and in treatment-resistant settings (in both cell lines and primary samples), hinting that bortezomib could have a significant role in the relapsed/refractory setting as well.^23, 26^

### Proteasome inhibition induces autophagy

Another mechanism by which cells can dispose of undesired protein products is through autophagy. Normally, autophagy acts as an adaptive and protective process that mitigates the deleterious effects of nutrient deprivation, growth factor withdrawal or metabolic stress.^31^ It involves the rearrangement of subcellular membranes to sequester organelles or proteins within autophagosomes and subsequent delivery of these species to the lysosome for degradation.^31^ Under conditions of extreme cellular stress, however, cells can instead use autophagy to degrade essential components and undergo cell death.^32^ Intriguingly, bortezomib-induced proteasome inhibition has been shown to induce autophagy in both primary AML samples and several cell lines (Figure 1).^31, 32^

Internal tandem duplications of the Fms-like tyrosine kinase 3 receptor (FLT3-ITD) are found in ∼20% of AML patients and are associated with poorer outcomes.^1^ Recent efforts to target FLT3 with the tyrosine kinase inhibitor quizartinib have demonstrated efficacy by inducing CRs in patients with refractory disease; however, point mutations within the kinase domain of FLT3 confer quizartinib resistance and have been detected in patients.^31^ Therefore, treatments that modulate FLT3 expression independent of kinase inhibition are of particular interest.

Larrue *et al.*^31^ showed that FLT3-ITD^+^ primary AML samples are more sensitive than wild-type samples to proteasome inhibition by bortezomib, and that this sensitivity correlates to *FLT3-ITD* allele burden. In this setting, bortezomib treatment led to inhibition of mammalian target of rapamycin complex 1 (mTORC1, a potent inhibitor of autophagy), increased conversion of LC3-I–LC3-II (a marker of autophagosome formation), and ultimately, autophagy of FLT3 protein.^31^ Furthermore, autophagy of FLT3 occurred in both quizartinib-sensitive and resistant cells lines, indicating that bortezomib treatment may represent a therapeutic strategy in FLT3-ITD^+^ AML patients refractory to tyrosine kinase inhibition.^31^

TNF receptor associated factor 6 (TRAF6) is a key protein target of *miR-146a*, a candidate gene in del(5q) MDS/AML that is reduced significantly in both del(5q) and normal karyotype cases.^32^ Treating AML cell lines with bortezomib induced autophagy of TRAF6 and subsequent apoptosis.^32^ Although TRAF6 overexpression conferred some resistance to bortezomib, sensitivity could be rescued by RNAi-mediated knockdown of *TRAF6*.^32^ Subsequent gene-expression analyses found that TRAF6 regulates the expression of the *PSMA1* gene, which encodes the α1 subunit of the proteasome.^32^ This demonstrates that while TRAF6 is regulated by bortezomib-induced autophagy, it also alters sensitivity to the drug by controlling *PSMA1* expression.^32^

### Mechanisms of resistance to proteasome inhibition

Unsurprisingly, several mechanisms of resistance to the cytotoxic effects of proteasome inhibition have been noted. First, cells (including LSCs) can overcome the effects of bortezomib by upregulating levels of NF-κB and accumulating the anti-apoptotic proteins MCL-1 and Bim. In these cases, co-administration of mechanistically relevant agents, such as the MCL-1 inhibitor obatoclax, re-sensitized the cells to bortezomib treatment.^22, 33^ Second, overexpression of proteasome subunits, such as PSMA1, confers resistance to bortezomib.^32^ Here, knockdown of upstream elements controlling the expression of PSMA1, including TRAF6, restored sensitivity in drug-resistant cell lines.^32^ Third, some *in vivo* data suggests a physical barrier to bortezomib may exist. In an *Mll*^*PDT/wt*^:*Flt3*^*ITD/wt*^ murine model – which develops spontaneous AML with phenotypic similarities to the human disease – bortezomib treatment alone did not produce a response *in vivo*, despite evidence of *ex vivo* efficacy.^34^ When encapsulated within liposomes, which is predicted to enhance tumor permeability and retention, bortezomib was able to induce a long-term disease-free remission in 80% of the mice.^34^

Another mechanism of resistance may be inherent to the drugs themselves. Some studies have suggested that bortezomib has limited efficacy in reducing the long-term culture-initiating cell (LTC-IC) frequency of some primary AML CD34^+^ cells, despite similar overall cytotoxic efficacy.^35^ In these cases, the second-generation proteasome inhibitor carfilzomib demonstrated superior reduction of LTC-IC frequency, perhaps due to the irreversible, more specific, and more prolonged inhibition of the proteasome. Congruent with other studies demonstrating the selectivity of proteasome inhibition, neither the LTC-IC frequency of normal CD34^+^ cells nor the colony-forming potential of normal bone marrow cells were significantly affected by carfilzomib exposure, implicating a leukemia-specific effect.^15, 35^ Finally, this study also showed that, similar to bortezomib, treatment with carfilzomib upregulates MCL-1 expression, conferring treatment resistance that could potentially be overcome by simultaneous administration of obatoclax.^35^

Encouragingly, expression of multi-drug resistance genes such as *Pgp*, *MRP-1*, *BRCP* and *LRP* seems to have little, if any, effect on the efficacy of proteasome inhibition with bortezomib in AML cell lines overexpressing these proteins.^26^

## Other roles of the proteasome in AML

### The proteasome as a mediator of anti-AML activity

Despite the promising prospect of proteasome inhibition in the treatment of AML, other therapeutic regimens rely upon a functioning proteasome for their efficacy. Nucleophosmin 1 (*NPM1*) gene mutations represent the most frequent genetic lesion in AML, present in ∼30% of cases.^1^ In primary *NPM1*-mutated AML cells, co-treatment with arsenic trioxide and all-*trans* retinoic acid – a regimen typically reserved for acute promyelocytic leukemia – was shown to induce proteasome-dependent degradation of NPM1 leukemic protein and induce apoptosis.^36^ Furthermore, this degradation of NPM1 was shown to potentiate the response to daunorubicin, which was unexpected considering that proteasome inhibition has been shown to be synergistic with anthracycline therapy in other cases of AML.^15, 26, 36^

### The proteasome as a clinical marker

Both the enzymatic activity and sheer quantity of the constitutive core particle (cCP) of the proteasome have been examined as potential clinical markers for a variety of outcomes, including sensitivity to treatment regimens and for OS. Matondo *et al.*^37^ investigated the sensitivity of several AML cell lines and primary patient samples to proteasome inhibitors and demonstrated that higher apoptotic responses were directly correlated to increased expression of the cCP. Of note, cCP expression was significantly higher in AML cells than in normal control cells, conferring cytotoxic selectivity for the treatment.

Circulating proteasomes behave similarly to and most likely reflect the activity of cellular proteasomes in primary leukemia samples.^38^ To provide a more rapid clinical assessment of proteasome status, Ma *et al.*^38^ sought to correlate levels of circulating proteasome complexes to a variety of clinical and laboratory data. They showed that the levels and cumulative activity of circulating proteasomes were significantly higher in AML patients than healthy controls; however, when the proteasome activity was normalized to the levels of proteasome protein in the samples, the chymotrypsin-like (ChTL) unit activity was lower in AML samples. This result correlates to *in vitro* work showing that KG1a AML cells, which are highly sensitive to bortezomib treatment, overexpress the 20S cCP but have lower ChTL activity due to altered expression of the 19S regulatory complex.^37^ Therefore, the increased activity of circulating proteasomes is likely a measure of leukemic tumor burden rather than enhanced catalytic efficiency. Indeed, levels of circulating proteasomes were predictive of ubiquitin levels, lactate dehydrogenase concentration and white blood cell count.^38^

AML patients with circulating proteasome levels below the median level (875 ng/ml) were found to have significantly better response rates to current treatment regimens than those with higher levels.^38^ Similarly, lower proteasome levels were a strong predictor of survival in AML patients with unfavorable and intermediate cytogenetics, and this remained true independent of other major risk factors examined (cytogenetics, age >70, and ECOG score>2).^38^ However, the duration of CRs did not correlate with either proteasome or ubiquitin levels.^38^ Ultimately, these studies suggest that circulating proteasome levels and enzymatic activities may prove to be useful biomarkers in risk-stratifying AML patients and in identifying patients who may be more likely to respond to proteasome inhibition.

## The immunoproteasome in AML

The immunoproteasome (i-prot) is an inducible proteasome primarily recognized for its role in antigen processing and presentation.^39^ Although its primary function is to degrade antigenic peptides for major histocompatibility complex class I (MHC-I) restricted antigen presentation, the i-prot has also been shown to influence T-cell polarization and differentiation, cytokine production by macrophages, degradation of proteins damaged by oxidative stress and NF-κB signaling.^40, 41^ As such, the i-prot is suspected to have a substantial role in the pathogenesis of a broad range of disorders, including inflammatory autoimmune diseases and hematologic malignancies.

**Figure 2:**
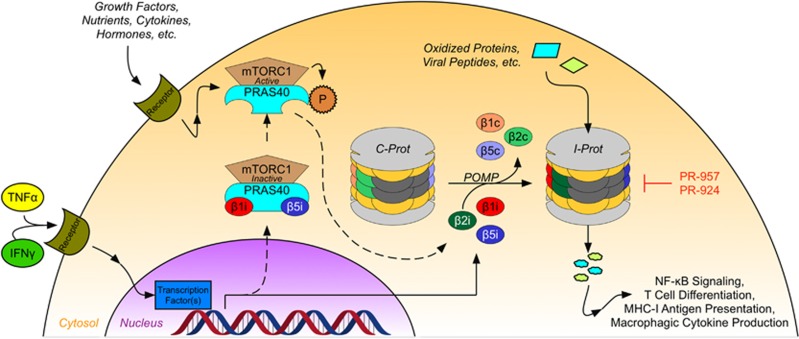
The i-prot has distinct features compared with the constitutional proteasome. The i-prot is formed when the β1c, β2c and β5c subunits of the c-prot are replaced by β1i and β5i, respectively. The expression of these subunits is promoted – at least in part – by IFNγ and TNFα signaling. Once expressed, β1i and β5i associate with the chaperone protein POMP, which facilitates their incorporation into the proteasome. In AML, however, it has been demonstrated that β1i and β5i are sequestered by PRAS40, a component of mTORC1. Once mTORC1 is activated by any of a number of methods (hyperactivation of mTORC1 is a feature of numerous malignancies), it phosphorylates PRAS40 and releases β1i and β5i, thus facilitating the assembly of the i-prot. The incorporation of these different subunits alters the proteolytic substrate selectivity of the i-prot, granting the i-prot distinct functions as compared with the c-prot.

In contrast to the constitutive proteasome (c-prot), which is tonically expressed in most cells, the i-prot is formed in response to the inflammatory cytokines TNF-α and interferon-γ (IFN-γ).^40, 41^ The i-prot is assembled when components of the c-prot are replaced by i-prot-specific subunits (Figure 2); these subunits shift the substrate and cleavage specificity relative to the c-prot,^3, 42^ suggesting that the i-prot degrades a selective subset of polyubiquitinated proteins.

Specifically, the core particle of the immunoproteasome (iCP) is formed when the three main catalytic subunits of the cCP – β1c, β2c and β5c – are replaced by subunits specific to the i-prot, β1i, β2i and β5i. These proteins are encoded by the genes *LMP2* (*PSMB9*), *LMP10* (*PSMB10*) and *LMP7* (*PSMB8*), respectively, and their expression is induced at least partly by TNF-α and IFN-γ.^3^ When incorporated into the iCP, these distinct subunits confer a different profile of substrate specificity that helps shape the diverging roles of the i-prot and c-prot; for example, the i-prot preferentially hydrolyzes proteins following non-polar amino acids, thereby generating peptide sequences with hydrophobic C-termini that are readily suited for incorporation into MHC-I complexes.^3^ Furthermore, the unique composition of the iCP has enabled the pre-clinical development of small molecule inhibitors specific for the iCP, which have served as valuable tools for probing the role of the i-prot in a variety of disease models.

The i-prot is an especially interesting drug target for the treatment of AML for several reasons. First, somatic mutations in genes encoding the iCP subunits – *LMP2* in particular – have been associated with an increased risk of developing AML, perhaps due to alterations in the substrate specificity or proteolytic activity of the complex.^43^ Second, the upregulation of total proteasome machinery that is observed in AML cells is characterized by a marked overexpression of the iCP, outweighing the cCP by approximately threefold.^44^ The ratio of β5i/β5c is even higher (7.9-fold).^44^ Third, there is recent evidence that the i-prot has a crucial role in mediating mTORC1-driven cell survival.^45^ Notably, mTORC1 hyperactivation is observed in many cell growth disorders, including AML. It was shown that PRAS40 (proline-rich Akt substrate 40 kDa), a component of mTORC1, binds to the β1i and β5i subunits and sequesters them to hinder iCP formation.^45^ Once phosphorylated by mTORC1, PRAS40 releases the iCP subunits, permitting their association with the chaperone protein POMP (proteasome maturation protein) and thereby facilitating i-prot assembly.^45^ In this manner, activation of mTORC1 serves to upregulate i-prot activity, enabling cells to deal with the defective ribosomal products that accumulate alongside increased protein synthesis and cell division. Furthermore, this mechanism has been validated in AML, thus defining a novel mechanism by which these cells protect themselves against protein stress and further elucidating a potentially fruitful therapeutic target.^45^

**Figure 3:**
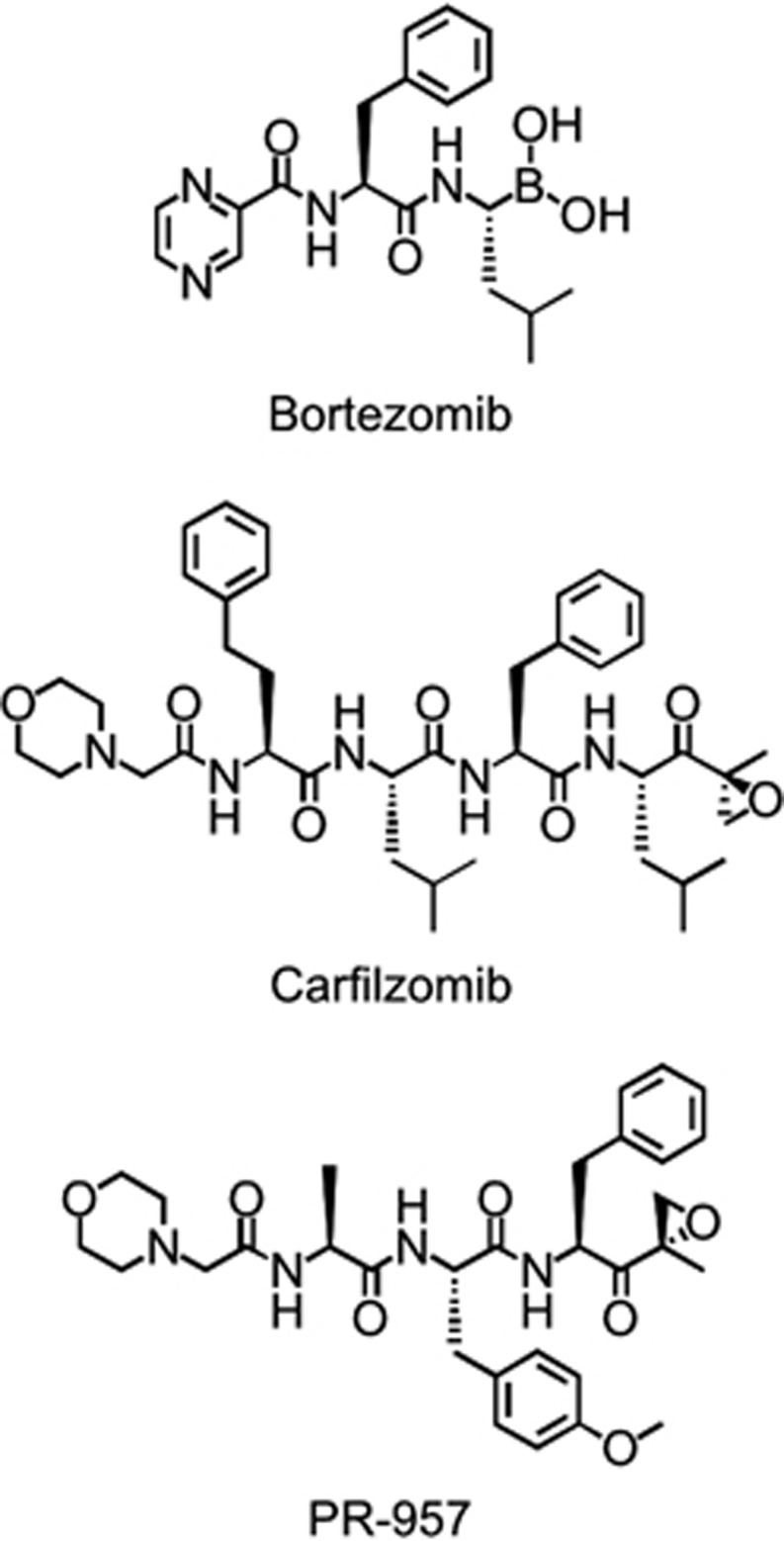
Structures of key proteasome inhibitors. Chemical structures of select clinical and investigational proteasome inhibitors: the dipeptide boronic acid bortezomib, tetrapeptide epoxyketone carfilzomib and tripeptide epoxyketone PR-957.

Perhaps the most widely used i-prot inhibitor is PR-957 (Figure 3, also known as ONX-0914), a tripeptide ketoepoxide-based covalent inhibitor in pre-clinical development by Onyx Pharmaceuticals (South San Francisco, CA, USA). Using *in vitro* preparations of human proteasome proteins, PR-957 was shown to selectively target β5i with up to 15-fold greater selectivity for the iCP than the cCP.^46^ Further, PR-957 was shown to inhibit β5i-specific antigen presentation and reduce the production of several cytokines, including interleukin (IL) 23, IL-6, IL-2, TNF-α and IFN-γ, thereby modulating the early activation of T cells and their differentiation into inflammatory effector cells.^46^ *In vivo*, PR-957 and a related ketoepoxide, PR-924, have shown efficacy in murine models of rheumatoid arthritis and MM, respectively, with both agents being well tolerated.^46, 47^

## Results from clinical trials with AML patients

With FDA-approved indications for MM and MCL plus pre-clinical efficacy in numerous other lymphoid malignancies, bortezomib has been incorporated into numerous clinical trial regimens either as a single agent or in combination with other drugs (Table 1). An early phase I study (NCT00005064)^48^ investigated the use of single-agent bortezomib in relapsed or refractory acute leukemias and enrolled a total of 15 patients, 11 of whom were diagnosed with AML. Bortezomib was administered intravenously twice weekly for 4 weeks every 6 weeks, with dose-limiting toxicities (that is, orthostatic hypotension, nausea, diarrhea and fluid retention) occurring at doses of 1.5 mg/m^2^. Proteasome inhibition was observed to be dose-dependent, reaching a maximum value of 68% at 1.5 mg/m^2^. This peak inhibition was observed 1 h after treatment, and proteasome activity returned to baseline over 72 h. In addition, peripheral blood mononuclear cells isolated from these patients underwent significant (>50%) apoptosis when exposed to bortezomib *in vitro*. Clinically, five patients met criteria for hematological improvement, four for a decrease in blast count (to ⩽5% in three patients) and one for an improvement in neutrophils. The primary diagnoses (that is, AML, ALL or MDS) for these patients were not reported.

In a phase II study (EUDRACT 2006-006923-38)^49^ including 14 patients with either untreated or relapsed/refractory AML, single-agent bortezomib was given at 1.5 mg/m^2^ twice weekly for 2 weeks in a 21-day cycle. A reduction of peripheral and/or bone marrow blasts was observed in 8 of the 13 evaluable patients (61%), and the median OS was 4 months (range 0.25–10 months). Peripheral neuropathy was the most frequently reported adverse event, experienced by 7 of the 13 patients (54%); 4 of these 7 (57%) had to discontinue treatment because of the neurotoxicity.

### Bortezomib plus anthracyclines

A phase I study (NCT00382954)^20^ administered bortezomib twice weekly at escalating doses (0.8, 1.0 or 1.2 mg/m^2^) alongside once weekly doses of idarubicin (10 mg/m^2^) for 4 weeks. Twenty patients were treated, 13 elderly individuals (median age 68 years) with newly diagnosed AML and another 7 with relapsed AML (median age 58). The study found the maximum tolerated dose (MTD) for this regimen to be 1.2 mg/m^2^ bortezomib plus 8 mg/m^2^ idarubicin, with common adverse events including neutropenic fever, infections, constitutional symptoms and gastrointestinal symptoms. Unlike the single-agent studies, no patients experienced neurotoxicities. One patient died due to treatment-related infection, and another four died from refractory AML while on the study. Ultimately, only four (20%) patients achieved a CR, though most (75%) did experience a hematologic response in the form of reduced peripheral blasts. The median OS in this high-risk group of patients was 4 months.

Another phase I trial^18^ of bortezomib plus pegylated liposomal doxorubicin (PegLD) enrolled 42 patients, five of whom were diagnosed with AML. Bortezomib was administered in a similar fashion as the single-agent trial and the same MTD of bortezomib was identified (that is, 1.5 mg/m^2^), likely due to the distinct mechanisms of action and lack of interactions between the two agents. Still, the dose-limiting toxicities observed were reminiscent of both agents, including cytopenias, fatigue, neuropathy and diarrhea. Of the five AML patients who enrolled, only two received two or more cycles of the treatment. The first of these two patients had newly diagnosed AML, but was not a candidate for standard therapies; they achieved a partial remission (PR) after two cycles, with a reduction in peripheral blood absolute blast count from 2,688 μl^−1^ (7%) at baseline to 107 μl^−1^ (1%). The second AML patient had relapsed disease post several induction regimens and an autologous hematopoietic stem cell transplantation; after two cycles, the hypercellular bone marrow with 19% blasts became normocellular with 4% blasts, though a clonal cytogenetic abnormality was still identified. This patient's treatment was then interrupted due to disseminated *Varicella zoster* reactivation, and upon resolution, the leukemia had progressed. Additional cycles of the bortezomib/PegLD regimen again converted their hypercellular marrow with 28% involvement to a normocellular marrow with only 5% blasts.

### Bortezomib plus hypomethylating agents

Building upon pre-clinical data,^29^ a phase I study (NCT00624936)^50^ investigated the combination of daily azacitidine (75 mg/m^2^) and escalating bortezomib (up to 1.3 mg/m^2^) in 23 adults with relapsed/refractory AML. Although these doses were reached without limiting toxicities, several patients did experience febrile neutropenia and/or infection. Five of the 23 patients (22%) achieved a remission, with two CRs and three CRs with incomplete recovery of platelets or neutrophils (CRi). Furthermore, four of these five responders presented with cytogenetic abnormalities (various karyotypes), and three of them achieved a cytogenetic CR.

Similarly, the combination of bortezomib and decitabine was also explored.^30, 51^ In this phase I trial (NCT00703300),^51^ 19 poor-risk AML patients (median age 70 years) were treated with an induction regimen of 10 mg/m^2^ decitabine intravenously on days 1–10. This was augmented with bortezomib escalated up to 1.3 mg/m^2^ on days 5, 8, 12 and 15. Although this dosing schedule was tolerable, bortezomib-related neuropathy was noted after repetitive cycles (none during cycle 1). Among the previously untreated patients, 5 of 10 (50%) achieved a CR (*n*=4) or CRi (*n*=1), while the overall CR/CRi rate was 7 of 19 (37%).

### Bortezomib plus histone deacetylase inhibitors

Despite pre-clinical efficacy, a phase II trial (NCT00818649)^52^ exploring the combination of bortezomib with the histone deacetylaseinhibitor vorinostat in high-risk patients with AML (*n*=8) or MDS (*n*=4) was terminated after three fatal cardiac events. The significant toxicity observed in this trial was quite unexpected given the favorable profile of similar studies in patients with MM and MCL. However, this patient population was heavily pretreated with a median of three prior regimens, and over half were relapsed after allogeneic hematopoietic stem cell transplantation. Although performance statuses were reasonably high, the extensive pretreatment may have affected the patients' ability to tolerate further toxicity. In the end, only one patient – an AML patient who had failed prior induction therapy – achieved CR; they remained transfusion independent for ∼6 months before evidence of disease progression. All patients succumbed to progressive disease within 1 year.

### Bortezomib plus combination chemotherapy

Another phase I study (NCT00505700)^19^ investigated the combination of bortezomib with a standard of care induction regimen (idarubicin plus cytarabine). A total of 31 patients were enrolled, 9 with relapsed AML and the other 22 with previously untreated AML. Non-hematologic toxicities included hypoxia, hyperbilirubinemia, transaminitis, fatigue and diarrhea. Overall, 19 (61%) patients achieved a CR, another 3 (9%) achieved a CR without recovery of platelets (CRp) and an additional 2 (6%) experienced a partial response (PR). For the 22 patients who achieved CR/CRp, the median disease-free survival was 15.3 months and the median OS was 17.6 months.

In a phase II study (NCT00742625)^28^ by the same investigator, the combinatorial use of bortezomib plus daunorubicin and cytarabine in 95 adults with previously untreated AML was examined. The results were similar, with 62 of 95 patients (65%) achieving a CR and an additional 4% achieving a CRp. Significant (grade 3 or greater) neuropathy was reported in 11 (12%) patients. Median disease-free survival was 8 months, whereas OS was 12 months.

Bortezomib was also examined alongside the tyrosine kinase inhibitor midostaurin plus mitoxantrone, etoposide and cytarabine (MEC) in a phase I trial (NCT01174888).^53^ In 23 relapsed/refractory AML patients who received this five-drug regimen, the overall CR/CRi rate was 82.5%, with 13 patients (56.5%) achieving CR and another 6 (26%) achieving CRi; the median OS for all patients was 330 days. Treatment-related toxicities were as expected, including peripheral neuropathy, decreased ejection fraction and diarrhea.

Again, not all trials have been successful. A phase II study (NCT00666588)^54^ of bortezomib combined with one of two reinduction chemotherapy regimens (either idarubicin/cytarabine or cytarabine/etoposide) in children with relapsed, refractory, or secondary AML was terminated due to failure to reach predetermined efficacy thresholds. However, this may be due to how the study defined its efficacy goals. For instance, only CR and CRp were considered efficacious responses, whereas CRi (incomplete recovery of blood counts, in this case) was excluded. Because the number of patients achieving a CRi (*n*=5) was similar to those achieving a CRp (*n*=3), this may have skewed the results at the interim analysis and led to the early trial cessation. In this study, overall CR rates reached 57% with a two-year OS of 39±15%. Correlative studies in this population revealed that depletion of leukemia-initiating cells in the bone marrow after one cycle of treatment was significantly associated with a clinical CR.

### Bortezomib plus other agents

Lenalidomide is an immunomodulatory agent with anti-angiogenic properties that has demonstrated efficacy in MM, MDS and MCL.^55, 56, 57, 58^ A phase I dose escalation study (NCT00580242)^59^ evaluated the MTD of bortezomib (intravenously on days 1, 4, 8 and 11) when added to standard dose lenalidomide (10 mg/day PO for the first 21 days) in a 28 day cycle. Twenty three patients were enrolled, nine of whom had AML (other 14 had MDS). The MTD of bortezomib in this regimen was 1.3 mg/m^2^, and among the nine AML patients, there was one CR and a median OS of 5.3 months.

The heat shock protein 90 (HSP90) inhibitor 17-allylamino-17-demethoxygeldanamycin (17-AAG) has demonstrated pre-clinical efficacy in AML, exhibiting cytotoxic effects in AML cell lines harboring *FLT3* or *BCR-ABL* mutations and inducing apoptosis in primary AML cells.^60, 61, 62^ In a phase I study (NCT00103272)^63^ of 17-AAG plus bortezomib in 11 patients with relapsed/refractory AML, the MTDs were 150 mg/m^2^ and 0.7 mg/m^2^, respectively. Hepatic and cardiac toxicities were dose limiting, and no clinical benefit was observed.

### Current trials and conclusions

Overall, the clinical use of proteasome inhibitors in AML appears to offer favorable outcomes, especially when combined with other combination chemotherapy regimens. As such, there are several currently active and recruiting trials continuing to explore the role of proteasome inhibition in AML (Table 2). Although much of the data available at this point is derived from studies of bortezomib, trials of the second-generation proteasome inhibitor carfilzomib are ongoing (for example, NCT01137747), and these results may provide additional insight into how best to modulate proteasome activity in this disease. Finally, human trials of i-prot-specific inhibitors are on the horizon and hope to offer enhanced anti-leukemic cytotoxicity, especially the depletion of LSCs, while minimizing the common adverse effects of pan-proteasome inhibition.

## Conclusions

These studies highlight the important role of the proteasome in AML biology and suggest that proteasome inhibition may be an effective therapeutic option in AML. To better understand the molecular effects and clinical outcomes of proteasome inhibition, further investigation is needed is several areas.

First, the eradication of the primitive LSCs has been a goal of the field since their elaboration over two decades ago.^64^ As proteasome inhibitors have demonstrated cytotoxic efficacy against LSCs, further study regarding how this approach can be targeted towards these cells may lead to more efficacious clinical use of these agents.^13, 14, 15^ One potential avenue for accomplishing this is through selective inhibition of the i-prot. Given that somatic mutations within the i-prot have been associated with an increased risk for developing AML and that iCP subunits are robustly overexpressed in primary AML cells, the i-prot is an attractive target for further drug development.^43, 44, 45^ Whether or not i-prot inhibition is more efficacious than c-prot inhibition (and whether any efficacy holds for LSCs), however, remains to be seen. Third, further work is needed to identify more rational drug combinations based upon known AML biology. For example, the addition of the MCL-1 inhibitor obatoclax to bortezomib was able to overcome resistance to proteasome inhibition and restore sensitivity to this agent.^33^ Likewise, there is evidence suggesting that a similar approach would be efficacious with the second-generation proteasome inhibitor carfilzomib.^35^ Finally, the identification of leukemia subtypes in which proteasome inhibition is likely to have a beneficial effect is of high priority. Like most therapies, it is unlikely that proteasome inhibition will be universally efficacious, so the ability to rapidly identify patients who will benefit from this intervention would be immensely helpful and enable the responsible use of these agents.

Ultimately, the proteasome has proven to be a highly interesting and potentially useful therapeutic target for AML. Current work has begun to elaborate its specific role in the biology of the disease, and the clinical outcomes of proteasome inhibition are encouraging.

## Figures and Tables

**Table 1:** Completed clinical trials of proteasome inhibition in AML patients

*Trial no. (Phase)*	*Patients (Median age)*^a^	*Intervention*	*Adverse events*^b^	*Response*^c^	*Survival (Months)*^d^
NCT00005064 (I)	15 total, 11 r/r AML (59 years)	Bortezomib	Orthostatic hypotension Nausea Diarrhea	No CR/CRi Reduced blasts in 33%	N/R
EUDRACT 2006-006923-38 (II)	14 new or r/r AML ineligible for conventional chemotherapy (70 years)	Bortezomib	Infection/FN Neuropathy	No CR/CRi Reduced blasts in 61%	4
NCT00382954 (I)	20 new or r/r AML (65 years)	Bortezomib Idarubicin	Infection/FN Constitutional Gastrointestinal	20% CR 5% PR Reduced blasts in 75%	4
NCT00624936 (I)	23 r/r AML (65 years)	Bortezomib Azacitidine	Infection/FN Neuropathy Thrombocytopenia	22% CR/CRi	N/R
NCT00703300 (I)	19 poor-risk AML (70 years)	Bortezomib Decitabine	Infection/FN Neuropathy	37% CR/CRi	N/R
NCT00818649 (II)	12 total, 8 AML (65.5 years)	Bortezomib Vorinostat	Cardiac events Nausea Neuropathy	8% CR	N/R^e^
NCT00505700 (I)	31 new and r/r AML (62 years)	Bortezomib Idarubicin Cytarabine	Hypoxia Hyperbilirubinemia Elevate AST	71% CR/CRi	12
NCT00742625 (II)	95 untreated AML (67 years)	Bortezomib Daunorubicin Cytarabine	Infection/FN Neuropathy	69% CR/CRi	12
NCT01174888 (I)	23 r/r AML (53 years)	Bortezomib Midostaurin MEC	Neuropathy Decreased LVEF Diarrhea	82.5% CR/CRi	11
NCT00666588 (II)	37 r/r AML (8 years)	Bortezomib plus: Idarubicin/Cytarabine *or* Cytarabine/Etoposide	Infection/FN Hypokalemia Hypoxia	57% CR/Cri *or* 48% CR/CRi	39% at 2 years
NCT00666588 (I)	23 total, 9 AML (73 years)	Bortezomib Lenalidomide	Infection/FN Neuropathy Hypoxia	11% CR/CRi	5
NCT00103272 (I)	11 r/r AML (63 years)	Bortezomib 17-AAG^f^	Infection/FN Hepatotoxicity Cardiotoxicity	No response	N/R

a R/r, relapsed/refractory.

b FN, febrile neutropenia.

c CR, complete remission, CRi, complete remission with incomplete recovery of platelets or neutrophils, PR, partial remission.

d N/R, not reported.

e No quantitative survival data was reported in this study, though all patients died of progressive disease within 1 year.

f 17-AAG, 17-allylamino-17-demethoxygeldanamycin.

**Table 2:** Active clinical trials of proteasome inhibition in AML patients

*Trial no.*	*Phase*	*Patients*^a^	*Intervention*
NCT01861314	I	Poor-risk or r/r AML	Bortezomib Sorafenib Decitabine
NCT02312012	I	Relapsed AML/MDS post allogeneic HSCT	Bortezomib Lenalidomide
NCT02352558	I	Advanced or r/r hematologic malignancies (inc. AML)	Bortezomib Napabucasin (BBI608)
NCT01137747	I	R/r AML and ALL	Carfilzomib
NCT01534260	I/II	Complex, poor-risk, or FLT3-ITD^+^ AML	Bortezomib Vorinostat Sorafenib
NCT02419755	II	MLL^b^ rearranged r/r hematologic malignancies (inc. AML), age ⩽21 years	Bortezomib Vorinostat
NCT01736943	II	R/r AML	Bortezomib Pegylated liposomal doxorubicin
NCT01420926	II	New AML, age ≥60 years	Bortezomib Decitabine
NCT01371981	III	New AML, age <30 years	Bortezomib Sorafenib

a R/r, relapsed/refractory.

b MLL, mixed-lineage leukemia.
